# Investigating the Impact of Whole-Genome Duplication on Transposable Element Evolution in Teleost Fishes

**DOI:** 10.1093/gbe/evae272

**Published:** 2024-12-23

**Authors:** Rittika Mallik, Dustin J Wcisel, Thomas J Near, Jeffrey A Yoder, Alex Dornburg

**Affiliations:** Department of Bioinformatics and Genomics, University of North Carolina at Charlotte, Charlotte, NC, USA; Department of Molecular Biomedical Sciences, North Carolina State University, Raleigh, NC, USA; Department of Ecology & Evolutionary Biology and Peabody Museum, Yale University, New Haven, CT, USA; Department of Molecular Biomedical Sciences, North Carolina State University, Raleigh, NC, USA; Department of Biological Sciences, Genetics and Genomics Academy, and Comparative Medicine Institute, North Carolina State University, Raleigh, NC, USA; Department of Bioinformatics and Genomics, University of North Carolina at Charlotte, Charlotte, NC, USA

**Keywords:** living fossil, teleost genome duplication, transposable elements, Actinopterygii, Teleostei

## Abstract

Transposable elements (TEs) can make up more than 50% of any given vertebrate's genome, with substantial variability in TE composition among lineages. TE variation is often linked to changes in gene regulation, genome size, and speciation. However, the role that genome duplication events have played in generating abrupt shifts in the composition of the mobilome over macroevolutionary timescales remains unclear. We investigated the degree to which the teleost genome duplication (TGD) shaped the diversification trajectory of the teleost mobilome. We integrate a new high coverage genome of *Polypterus bichir* with data from over 100 publicly available actinopterygian genomes to assess the macroevolutionary implications of genome duplication events on TE evolution in teleosts. Our results provide no evidence for a substantial shift in mobilome composition following the TGD event. Instead, the diversity of the teleost mobilome appears to have been shaped by a history of lineage-specific shifts in composition that are not correlated with commonly evoked drivers of diversification such as body size, water column usage, or latitude. Collectively, these results provide additional evidence for an emerging perspective that TGD did not catalyze bursts of diversification and innovation in the actinopterygian mobilome.

**Significance**

We investigate the role of the teleost genome duplication on transposable element (TE) diversification in ray-finned fishes by integrating an analysis of the mobilome from a newly sequenced genome from *Polypterus bichir* with analyses from over 100 ray-finned fish genomes. We reveal that teleost fish TE diversity depicts a signature of lineage-specific shifts rather than a major burst of novelty near the origin of teleosts, suggesting a complex, nuanced history of TE evolution. Our results are in line with emerging hypotheses concerning the impact of ploidy events on long-term TE evolution, providing critical context for future research into the genomic and evolutionary mechanisms influencing TE diversity across half of all living vertebrates.

## Introduction

Transposable elements (TEs) can account for over 50% of a vertebrate's total genome content (Sotero-Caio et al. 2017). Aptly dubbed “jumping genes,” TEs possess the astonishing ability to rearrange and reposition themselves within a given genome (McClintock 1984), through the use of two primary strategies for transposing. Class I retrotransposons replicate through a “copy and paste mechanism” involving an RNA intermediate. This RNA molecule is reverse transcribed back into a DNA copy, which is then seamlessly integrated into the genome, leaving the original template element untouched (Hickman and Dyda 2015). In contrast, class II DNA transposons use a “cut-and-paste” technique to excise themselves from their current location and relocate to an alternate genomic location (Muñoz-López and García-Pérez 2010). Comparative studies of vertebrate TEs have revealed substantial heterogeneity in the composition of class I and class II TEs between major clades of vertebrates (Chalopin et al. 2015). These studies have implicated changes in TE composition with altered gene regulation (Torres et al. 2021; Rech et al. 2022), evolutionary changes in genome size (Böhne et al. 2008; Naville et al. 2019; Wong et al. 2019), evolutionary novelties (Senft and Macfarlan 2021), changes in life history (Niu et al. 2019), and speciation (Ricci et al. 2018; Serrato-Capuchina and Matute 2018) to name but a few. While the past decade has yielded tremendous strides towards developing an understanding of the general hallmarks of TE evolution, the evolutionary fate of TEs following whole-genome duplication events is just beginning to emerge (Parisod et al. 2010; Rodriguez and Arkhipova 2018; Papon et al. 2023).

Genome duplication events have the potential to amplify genome complexity, with emerging evidence highlighting a possible role for duplication events enabling phenotypic evolution through duplicated genes (Moriyama and Koshiba-Takeuchi 2018). Studies within individual or closely related species that vary in their level of ploidy have revealed substantial modifications that can facilitate profound epigenetic repatterning within the domains of TEs. However, the impact of such changes on the mode of TE diversification remains unclear. For example, surplus gene copies can compensate for potential losses or modifications in expression caused by TE insertions, thereby facilitating extensive genomic modification through the actions of TEs. This redundancy hypothesis (Matzke and Matzke 1998) suggests a substantial shift in TE content and composition following a genome duplication event that continues as lineages diversify (Parisod et al. 2010). An alternate hypothesis argues that genome duplication events correspond to a transitory phase for a species that is characterized by diminished population size (Lynch 2007). This bottleneck hypothesis argues that as the efficacy of selection decreases, the likelihood of moderately deleterious TE insertions within nascent polyploid genomes becoming fixed is increased. Consequently, this hypothesis would expect a pulse of TE diversification coincident with a genome duplication event (Parisod et al. 2010). Recently, a hypothesis framed around the concept of “Hopeful Monsters” suggested that the balance between the deleterious and beneficial aspects of TE proliferation can lead to occasional beneficial mutations that can promote speciation and contribute to the emergence of new traits (Turner et al. 2021). In contrast to the previous two hypotheses, this hypothesis predicts that genome duplications would result in only a limited number of TE changes, not a substantial pulse of diversification. Given that these hypotheses represent corner cases on a continuum of possibilities and alternate hypotheses, empirical comparative genomic studies at different evolutionary timescales are key to understanding the implications of genome duplication events on the evolution of TEs.

Teleost fish offer an exemplar group for the study of TEs and an ancient genome duplication event. Over 99% of the over 35,000 living species of actinopterygians are teleosts, a clade that experienced a genome duplication event in its early evolutionary history (Brunet et al. 2006; Glasauer and Neuhauss 2014). Comprising half of all living vertebrate species, teleosts have successfully radiated across virtually all aquatic habitats, including swamps (Albert et al. 2020), abyssal ocean depths (Davis et al. 2014; Miller et al. 2022), polar and high altitude regions (Near et al. 2012; Dornburg et al. 2017; De-Kayne et al. 2022; Parker et al. 2022), and caves (Wen et al. 2022). In contrast, the three remaining extant ray-finned fish lineages—Holostei (gars and bowfin, 8 species), Acipenseriformes (sturgeon and paddlefish, 29 species), and Polypteridae (bichirs and ropefishes, 14 species)—did not undergo the teleost specific genome duplication event. Recent studies of holostean genomes have suggested alternative views on the evolutionary diversification of the teleost versus nonteleost actinopterygian mobilome. On the one hand, differences in the abundance of TE types between holosteans and model teleosts such as Zebrafish (*Danio rerio*) and Medaka (*Oryzias latipes*) (Braasch et al. 2016; Thompson et al. 2021; Mallik et al. 2023) raise the possibility of an evolutionary shift following a genome duplication event in teleosts. On the other hand, the diversity of TE superfamilies is similar between holosteans and teleosts, suggesting the TGD had a limited impact on the generation of novelty in the teleost mobilome (Chalopin and Volff 2017). Further comparative studies of the ray-finned fish mobilome that include a broad representative sampling of teleosts, holosteans, acipenseriforms, and polypterids are necessary to discern the impact of the TGD on the teleost mobilome. With the growing number of genomes of ray-finned fishes deposited in public data repositories, such studies are now possible.

Over 20 years ago, the *Takifugu rubripes* genome represented the first ray-finned fish genome sequenced (Aparicio et al. 2002). Decreased costs of sequencing have since led to a surge of efforts to sequence additional actinopterygian genomes (Fan et al. 2020; Randhawa and Pawar 2021) that now present a wealth of resources for phylogenetically comprehensive comparative studies. In addition to the availability of hundreds of teleost genomes, there are genomes of a few for nonteleost ray-finned fishes. For example, the Sterlet (*Acipenser ruthenus*) genome revealed an independent historic genome duplication in this chondrostein lineage that provides an additional ancient duplication for understanding evolutionary patterns of TE diversification in actinopterygians (Cheng et al. 2019). Similarly, the sequencing of the Senegal Bichir (*Polypterus senegalus*) leveraged the anatomy of polypterids to provide critical insights into how vertebrates achieved the transition from water to land (Bi et al. 2021). In addition, the recently sequenced genome of a second polypterid, *Erpetoichthys calabaricus* (Reedfish; Assembly [ErpCal1.1; NCBI Annotation Release 100]), has been deployed alongside the *P. senegalus* genome to provide further insights into other aspects of early vertebrate diversification that include the evolution of keratins (Kimura and Nikaido 2021), olfactory receptors (Zhang et al. 2021), and numerous other traits (Helfenrath et al. 2021; Mikami et al. 2022). However, based on BUSCO scores, the currently sequenced genome of *P. senegalus*, remains incomplete. As the only *Polypterus* genome sequenced, the lack of an additional *Polypterus* genome challenges interpretation of results concerning the distribution of TEs in this lineage. Given the phylogenetic position of polypterids, additional genome sequencing efforts are of extreme value for contextualizing the diversification of ray-finned fish TEs.

Here we present a high-quality chromosome-level assembly and annotation for an additional *Polypterus* species, *Polypterus bichir*. We integrate analyses of TEs within this genome with data on TE content for all other major lineages of actinopterygians to investigate the impact of genome duplication on the early evolution of the teleost mobilome. Using a comparative phylogenetic framework, we test for associations between genome size and the composition of the mobilome, variations in mobilome composition between teleosts and nonteleost ray-finned fish lineages, as well as possible correlations between TE content and aspects of actinopterygian biodiversity such as habitat, body size, or water column occupation. We additionally reconstruct the ancestral mobilome of actinopterygians through the TGD event and assess the phylogenetic signal of class I and class II TEs. These results support an emerging perspective on the early diversification of the actinopterygian mobilome and the impact of the TGD on its evolution.

## Results and Discussion

### Bichir Genomes: an Example of Evolutionary Conservation or Recent Divergence?

We present a high-quality assembly of the *P. bichir* genome (NCBI Bioproject PRJNA811142). The results of a BLAST search of mitochondrial COI from our specimen against polypterid barcode sequences on NCBI verified the identification as *Polypterus bichir lapradei*, a currently not recognized subspecies based on morphology that has been suggested to represent a genetically distinct lineage (Near et al. 2014). The 10X supernova assembly from Dovetail Genomics (Scotts Valley, CA) resulted in 130,773 scaffolds forming a total final genome size of 3,962,089,718 bp. Here 13.9% of the genome (550,533,170 bp) is composed of the ambiguous base “N” and a GC content of 39.21%. During Dovetail Hi-Rise assembly, the input assembly was further incorporated into 70,587 longer scaffolds. The total length of the resulting Dovetail Hi-Rise assembly was 3,905.43 Mbp, with a contig N_50_ of 37.39 kbp. The N_50_ of the assembly was 202.693 Mbp scaffolds with a L_50_ of seven scaffolds. This is similar to the *E. calabaricus* genome which is 3.6 Gb long, with a contig N_50_ size of 6.8 Mb, and a scaffold N_50_ size of 217.7 Mbp, and the *P. senegalus* reference genome, which is 3.7 Gbp, with a scaffold N_50_ of 189.69 Mbp, and contig N_50_ of 4,528.14 kbp (supplementary table S1, Supplementary Material online). However, a comparison of the BUSCO analysis on the *P. senegalus* reference genome to the newly sequenced *P. bichir* genome reveal a striking difference between the two assemblies (supplementary table S2 and fig. S1, Supplementary Material online). We find nearly triple the number of actinopterygian orthologs in the *P. bichir* genome relative to the *P. senegalus* genome (supplementary fig. S1, Supplementary Material online). This suggests that the *P. bichir* sequence may fill additional gaps in our understanding of the genomic evolution of polypteriforms outside of the mobilome elements discussed in this study.

Contrasting patterns of synteny between our new sequenced genome and those of *P. senegalus* and *E. calabaricus* using D-genies supports a high level of synteny between these species (supplementary fig. S2, Supplementary Material online). Further, the number of *P. bichir* superscaffolds (18) reflects the described karyotypes of most polypterids: *E. calabaricus* (*n* = 18)*, Polypterus palmas* (*n* = 18)*, Polypterus delhezi* (*n* = 18), *P. senegalus* (*n* = 18), and *Polypterus ornatipinnis* (*n* = 18) (Bachmann et al. 1972; Vervoort 1980; Morescalchi et al. 2007; Krysanov and Golubtsov 2014). The only exception to this chromosome count known in polypterids is in *P. weeksi* (*n* = 19) (Vervoort 1980), the sister lineage to *Polypterus ornatipinnis*. While closely related vertebrate taxa can often diverge substantially in their TE content over time, we find that this is not the case in bichirs. Instead, the conservation of karyotype and synteny across these species is also reflected in the relative abundances of TEs across these species. For *P. bichir*, RepeatModeler quantified 53.40% of the genome to be composed of TEs. This is similar to *P. senegalus* with (54.66%) and *E. calabaricus* (60.35%) and overall posits a surprising level of genomic conservation between species of polypterids. This conservation could be a consequence of multiple factors including slow rates of molecular evolution such as those observed in gars and bowfin (Wright et al. 2022), the possible geologically recent Early Miocene crown age of Polypteridae estimated using molecular clocks (Near et al. 2014), or a combination of the two to name but one set of possibilities. Indeed, Kimura distances of *P. bichir* TEs (supplementary fig. S28, Supplementary Material online), reveal that the bichir genome is dominated by recent copies of mostly DNA and LINE transposons (*K* ∼ 5). Regardless of the mechanism, our sequencing of the *P. bichir* genome reveals polypterids as a candidate lineage for future studies of the mechanisms that promote genomic stability within a clade.

### The Evolution of the ray-finned Fish Mobilome

**Fig. 1.**
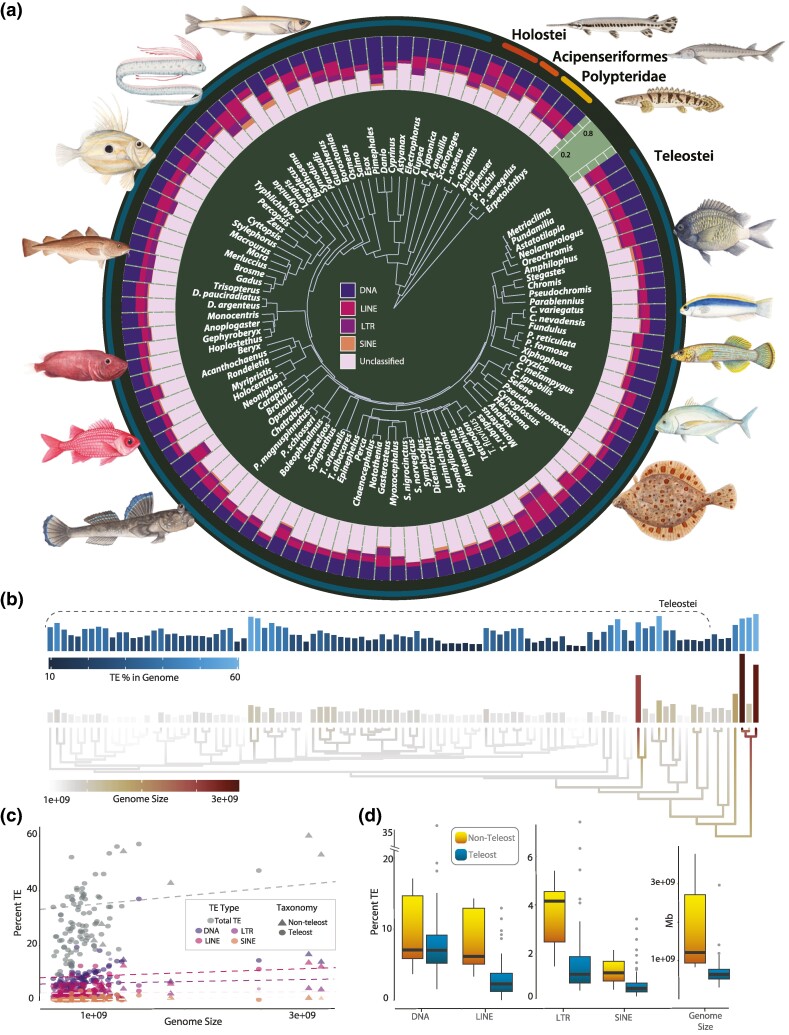
Major patterns of TE evolution across the evolutionary history of ray-finned fishes. a) The relative abundance of DNA, LTR, LINE, SINE transposons, and unknown transposons relative to actinopteryigiian phylogeny are shown. b) Total TE % and absolute genome size in the context of evolutionary history are compared. c) Results of a phylogenetic generalized least-squares regression to assess the relationship between genome size and TE abundance are provided. d) TE % and genome size between teleosts and nonteleost actinopterygians are compared. Shadings in (a) correspond to elements labeled in the panel. Shadings in (b) correspond to high (light or dark) and low values (dark or light) in the upper and lower panels, respectively. Shadings in (c) correspond to the labels in (a) for individual elements. Panels in (d) correspond to the quantiles of each category with dark horizontal lines indicating the mean value.

Placing our characterization of the polypterid mobilome into the context of other major ray-finned fish lineages reveals a complex history of mobilome evolution over the past 400 million years (Fig. 1a). The reconstructed phylogenetic history of TE evolution in actinopterygians is not consistent with a sudden TE proliferation coincident with the TGD event. Instead, our analyses reveal that compositional TE patterns at this scale largely reflect shifts within actinopterygian subclades. For example, cichlids have similar compositional patterns relative to pufferfish, the latter of which exhibits an increase in the number of LINE elements (Fig. 1a). Independently, Lampriformes (*Regalecus* & *Lampris*) also exhibit an expansion of their LINE elements to a relative level similar to that observed in pufferfishes (Fig. 1a). The ubiquity of such patterns of clade-specific heterogeneity are strongly supported by tests of phylogenetic signal using both Pagel's lambda (*λ*) and Blomberg's *K* (*K*) (supplementary table S3, Supplementary Material online). In all cases, *K* values are significant (DNA | *K* = 0.478, *P*  *=*  *6e-04*; LTR | *K*  *=* 0.405*, P*  *=*  *1e-04;* LINE | *K*  *=* 0.945*, P*  *=*  *1e-04*; and SINE | *K*  *=* 0.529*, P*  *=*  *3e-04*). Likewise, quantifications of lambda values for DNA (*λ*  *=*  *0.98, P*  *=*  *1.89e-13*), LTR (*λ*  *=* 0.819*, P*  *=*  *6.64e-11*), LINE (*λ*  *=* 0.975*, P*  *=*  *1.51e-19*), and SINE (*λ* ∼ 1.0*, P*  *=*  *4.37e-15*) are very close to 1, indicating strong phylogenetic signal, supporting a pattern of similar relative TE abundances between closely related taxa and increasing disparity between subclades.

Contrasting relative TE content (Fig. 1b) against total genome size reveals numerous expansions and contractions of both genome size and relative TE abundances across the phylogenetic diversity of ray-finned fishes. Importantly, there is no signal of a shift in the rate of TE evolution with the origin of teleosts. Instead, the reconstructed history indicates that expansions and contractions of both relative TE abundance and genome size appear to heterogeneously occur across various teleost and nonteleost lineages. Phylogenetic regression is essential when comparing data between species because it explicitly models the covariance structure arising from shared ancestry, accounting for the statistical dependence between species that results from their evolutionary relationships. Our phylogenetic regression analyses indicate that shifts in genome size are weakly correlated with changes in the relative abundances of TEs (Fig. 1c; supplementary table S3, Supplementary Material online). This result is consistent with previous studies of ray-finned fish mobile elements that suggest a modest correlation between the relative abundance of TEs and genome size (Yuan et al. 2018; Reinar et al. 2023), and supports the suggestion that lineage-specific shifts in mobilome expansion and deletion events have clade specific effects in mobilome composition (Chalopin et al. 2015). Correspondingly, we find strong evidence that genome size in ray-finned fishes also exhibits a pattern of strong phylogenetic signal (*K*  *=* 0.659*, P*  *=*  *6e-04; λ*  *=* 0.717*, P*  *=*  *4.45e-15*), paralleling a major trend in genome size evolution. Lineages as disparate as angiosperms (Beaulieu et al. 2008; Alonso et al. 2015), *Drosophila* (Sessegolo et al. 2016), marine dinoflagellates (Lo et al. 2022), bacteria, and Archaea (Martinez-Gutierrez and Aylward 2022) exhibit strong phylogenetic signal in genome size evolution. Given that the relative abundance of TE content in a genome is weakly correlated with genome size in ray-finned fishes (Lehmann et al. 2021) Fig. 1d; and numerous other lineages (Chalopin et al. 2015), it is possible that the evolution of TEs exhibits a signature of strong phylogenetic signal across the Tree of Life (Dodsworth et al. 2015).

Multiple factors promote genome size evolution (Olmo et al. 1989), including hypothesized correlations between the molecular evolution of the mobilome and overall increases in genome size. For example, transposition rates generally exceed excision rates (Kelleher et al. 2020), enabling TEs to contribute to the enlargement of genomes and exert an influence on genome size evolution. However, whether the TGD event resulted in a shift in the mode of evolution between the teleosts and nonteleosts mobilome remains unclear. We tested for differences between teleosts and nonteleosts using a phylogenetic analysis of variance (ANOVA) (Garland et al. 1993). This test calculates a traditional ANOVA and then compares the observed F-value to a null distribution generated by simulating trait evolution on the phylogenetic tree under a scenario where the independent variable (*x*) has no effect on the dependent variable (*y*). This approach accounts for the phylogenetic relationships by distinguishing whether similarities in the dependent variable among species arise from the influence of the independent variable or from shared evolutionary history, thereby removing possible confounding effects arising from shared common ancestry by considering the dispersion of x across the phylogeny. Our phylogenetic ANOVA reveals no support for a significant difference in TE content between teleosts and nonteleosts (*P* = 0.06; Fig. 1d).

**Fig. 2.**
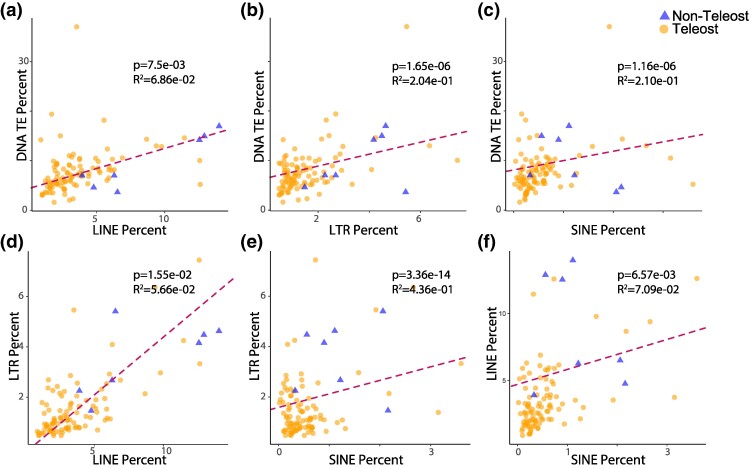
Correlation between relative class I and class II mobilome content. Panels a), b), and c) display the correlations between DNA transposons and LINE, LTR, and SINE transposons resulting from phylogenetic generalized least-squares regressions, respectively. Panels d) and e) illustrate the correlations based on a phylogenetic generalized least squares regression between LTR transposons and LINE and SINE transposons, respectively. Panel f) presents the correlation based on a phylogenetic generalized least-squares regression between LINE and SINE transposons.

Additionally, phylogenetic regression analyses strongly support a correlation between the content of all mobilome elements between species. When not accounting for differences in genome size, such a correlation may be expected if TEs only scale with genome size, and is indeed supported by our analyses (*P* < 0.0001 in all cases). However, when accounting for differences in genome size, our results continue to support that an increase or decrease in one element will also be correlated in an increase in the other for all TE types (Fig. 2). These results suggest that TE content is not just a function of genome size, as is also reflected in our finding of only a weak correlation between these variables (Fig. 1c). On the contrary, there is a growing body of evidence that TEs and the TE silencing machinery of a host are locked in an evolutionary arms race (Simkin et al. 2013). Of particular note are studies that have investigated how changes in DNA methylation sites can provide opportunities for TEs to become more promiscuous (Yoder et al. 1997; Chernyavskaya et al. 2017), including studies that have linked changes in DNA methylation to TE derepression that acts a post zygotic barrier during the speciation process (Laporte et al. 2019). Considering opportunities for subtle shifts in methylation and other mechanisms that inhibit or enable TE propagation across the teleost Tree of Life alongside our results, this suggest that the overall relative composition of the ray-finned fish mobilome has likely been shaped more by recent lineage-specific shifts in genome evolution than historical legacy stemming from the TGD event.

### Concerning the TGD and the Appearance of Novel Mobilome Elements

**Fig. 3.**
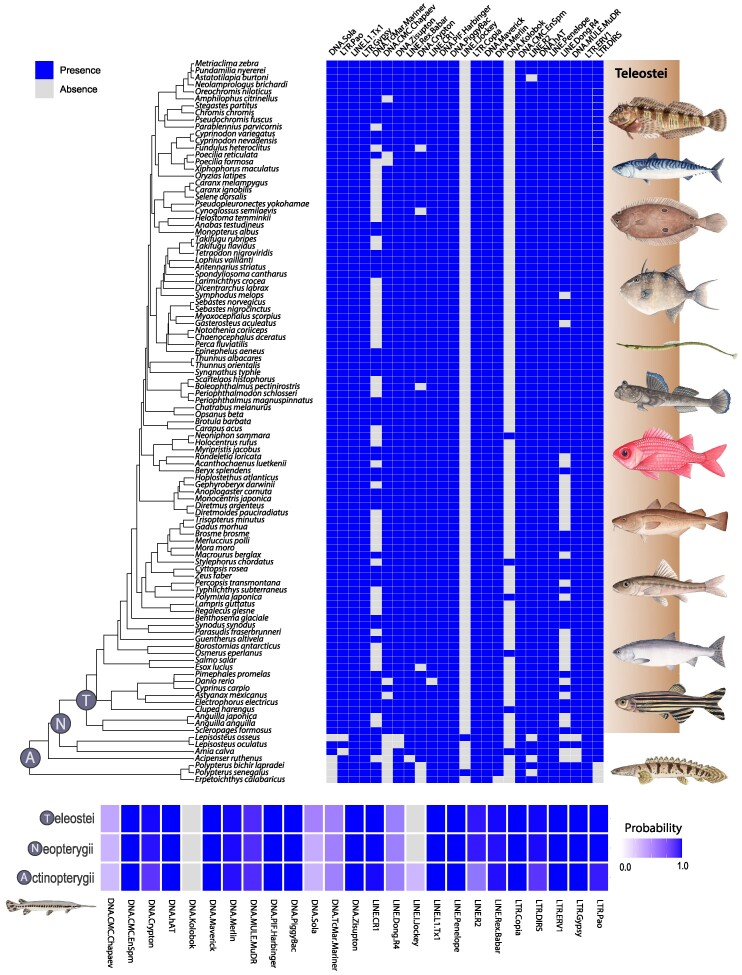
Reconstructing the ancestral actinopterygian mobilome. The presence and absence of TE superfamilies (listed above) are represented by blue and gray squares, respectively, for various species on the left (top panel). The shaded area behind the fish paintings represents teleosts, while the unshaded region below represents nonteleosts with evolutionary relationships between taxa depicted by the phylogenetic tree on the far-left. These input data were used to estimate the ancestral mobilome (the probability of each TE superfamily being present or absent) for the most recent common ancestor of all Actinopterygii (A), Neopterygii (N), or Teleostei (T) using SIMMAP (bottom panel). With a resulting heatmap plotted using ggplot (Wickham 2016).

It is possible that the TGD catalyzed the evolution of major groups of novel TE's. However, when placing the 25 main superfamilies of the teleost mobilome (Chalopin et al. 2015) into the context of the phylogenetic diversity of ray-finned fishes, we reveal that this effect was muted. We estimated the ancestral mobilome of actinopterygians utilizing a model-averaged stochastic character mapping approach (Revell 2024). Out of 25 superfamilies, the presence of 24 superfamilies observed in teleosts is mirrored within nonteleosts (Fig. 3). The only exception to this is the DNA transposon superfamily Chaepev (Kapitonov and Jurka 2007). It is unlikely that this superfamily arose as a consequence of the TGD, as it is present in several arthropods as well as a range of vertebrate species including anoles (Novick et al. 2011), and lampreys (Zhang et al. 2014). Chapaev sequences have also been documented from White Sturgeon (*Acipenser transmontanus*) (Zhang et al. 2014), suggesting that additional species of nonteleost ray-finned fishes possess this superfamily.

Genome duplication events are hypothesized to form a substrate for genomic innovation (Crow et al. 2006). In contrast to this expectation, our results reveal numerous instances of numerous likely TE losses as well as independent gains. For example, we find repeated losses of TE super families such as LINE Dong, DNA TcMariner, and DNA Crypton in teleosts (Fig. 3). LTR-Pao is absent in longnose gar and bowfin, suggesting a loss in holosteans. LTR-DIRS are absent in all the three polypterids (*P. bichir, P. senegalus,* and *E. calabaricus*). In addition, we identify the TE superfamily Jockey, a LINE element, exclusively in *Lepisosteus osseus* and *P. senegalus*. Jockey has been previously confirmed in coelacanth (Chalopin et al. 2014) and lampreys, and this marks the first reported instance of its presence in actinopterygians. It is possible that this element has been lost multiple times independently, however, Jockey is known to have high rates of horizontal transfer in other lineages (Reiss et al. 2019; Tambones et al. 2019), raising the possibility that these were independent gains.

Studies of vertebrate genome evolution often assume vertical transmission as the dominant mode of evolution. However, recent work has highlighted the impact of horizontal transfer events in the evolution of the vertebrate mobilome (Zhang et al. 2020; Galbraith et al. 2022; Paulat et al. 2023). Our ancestral mobilome reconstructions of the DNA Kolobok superfamily are in line with phylogenetic patterns expected by a model of horizontal transfer (Fig. 3). In our sampling, the presence of Kolobok is limited to five distantly related teleost lineages, as well as the Longnose.

Gar (*L. osseus*) among nonteleost ray-finned fishes. It is possible that the Kolobok element has been repeatedly transferred throughout the evolutionary history of Actinopterygii. Such transfer events are considered more likely by recent observations of horizontal transfer at more recent time scales. For example, there is evidence of horizontal gene transfer of DNA transposons like Merlin, TcMariner, and PiggyBAC in salmonids (de Boer et al. 2007). Likewise, there has been evidence of horizontal transfer of Chapaev transposons in White Sturgeon, Pacific Bluefin Tuna (*Thunnus orientalis*) and Blue Catfish (*Ictalurus furcatus*) (Zhang et al. 2014). As the number of high-quality genomes for species of ray finned fishes continue to accumulate, future studies of the extent of horizontal transfer events across the ray-finned fish mobilome offer an exciting research prospect.

### Lineage-specific Expansions of the Teleost Mobilome

**Fig. 4.**
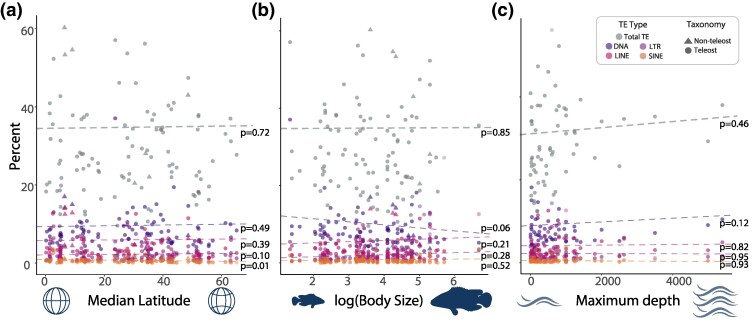
Correlation of TEs with biotic and abiotic factors. a) This panel displays the results of phylogenetic generalized least-squares (PGLS) regressions between TE percentages and median latitude b) This panel portrays the PGLS regression results between TE percentage and body size (log-transformed). Lastly, c) depicts PGLS results between TE abundance and the maximum habitat depth of the species. The colors and shapes of each data point on the plot are defined in the key.

TEs can play a beneficial role by enhancing an organism's ability to respond to dynamic environmental conditions (Yuan et al. 2018). Numerous studies have linked TE activity to an organism's responsiveness to environmental factors (Capy et al. 2000; Fujino et al. 2011; Hua-Van et al. 2011; Baduel et al. 2021). This association between TEs and environment suggests that there may be a correlation between relative TE abundance and specific aspects of an organism's ecological niche or life history. We tested this expectation using three commonly tested abiotic/biotic factors associated with evolutionary diversification: latitude, body size, and depth. However, we find no such association for several factors often invoked to explain diversification patterns at this evolutionary scale. Under the Brownian motion model, our phylogenetics least-squares regression (PGLS) analysis between TE content and maximum body size revealed no evidence for a strong correlation between these traits (Fig. 4a and supplementary tables S4 to S7, Supplementary Material online). Likewise, we find no statistically supported correlation between TE content and depth occupancy in marine fishes (Fig. 4b), in line with recent work suggesting that genome size and depth relationships have been driven by an association within just argentiniiforms, and that this is not a pattern across all teleosts (Medeiros et al. 2022). There is also no general pattern of a correlation between TE content and the average latitude of a species geographic distribution (Fig. 4c). The only exception to this lack of correlation between latitude and TE content occurs in SINEs (supplementary table S6, Supplementary Material online) under the Brownian motion model (*P* = 0.0197). This correlation is likely driven by a reduction in SINE elements in Antarctic notothenioids, which experienced an unusual bout of genomic evolution prior to their diversification in the Southern Ocean (Daane et al. 2019). Regardless, depth, body size, or latitude appear not to be predictors of mobilome evolution, even when differentiating between marine, freshwater, or estuarine fishes at this evolutionary scale. In all cases, phylogenetic regression results were largely similar between a brownian and Orstein–Uhlenbeck model of trait evolution (supplementary table S6 and S7, Supplementary Material online).

**Fig. 5.**
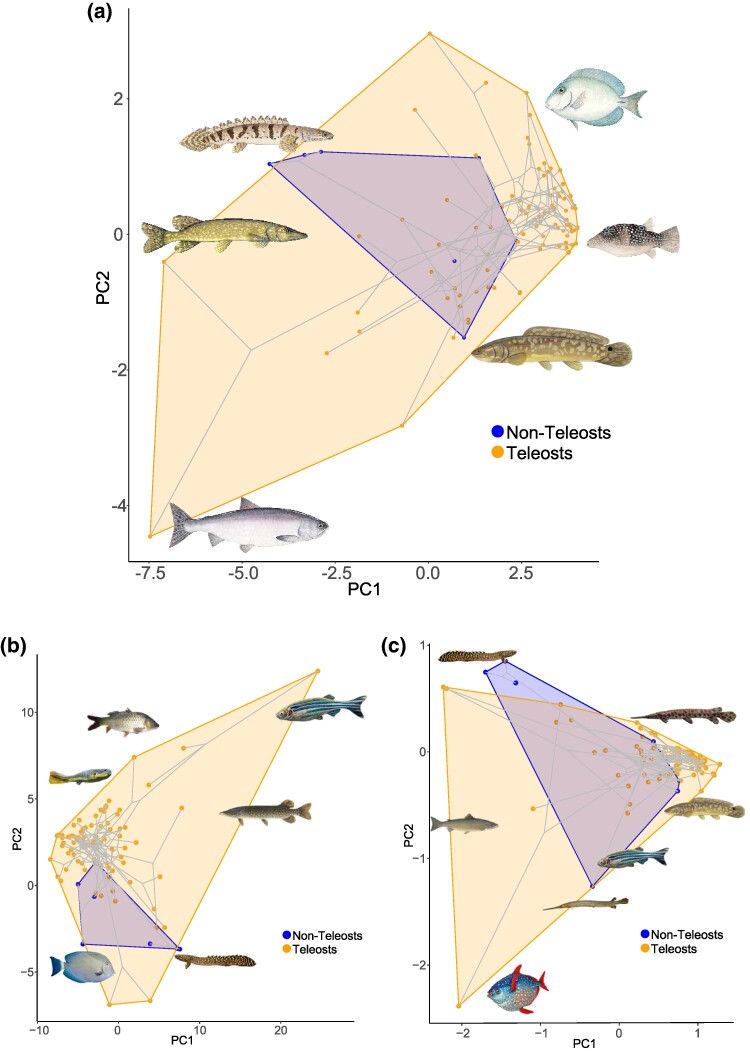
Considering TE diversity in the context of evolutionary history. a) Results of a Phylogenetic PCA on the abundances of LTRs, DNA transposons, LINEs, and SINEs that project the phylogeny and points onto a 2D plot of PC1 and PC2. b) Depicts the resulting space based on the residual variation following linear regression between major TE classes and genome size. c) Depicts the results of a PCA on the residual variation of the abundances of all 25 superfamilies accounting for differences in genome size, consisting of 13 DNA transposons, 7 LINEs, and 5 LTR subfamilies. Dark shading indicates nonteleost actinopterygians; light shading indicates teleosts. All the images of the fishes are from Wikimedia commons.

The TGD certainly could have presented opportunities for a burst of genomic novelty. However, the next 300 million years of ray-finned fish evolution would certainly be expected to shape the genomes of different lineages responding to different biotic and abiotic conditions. As such, it is possible that the signal of the genome duplication has eroded. For example, TEs could replace themselves in a way analogous to multigene families, in which recently diverging paralogs often replace older genes. In such a scenario, rapid rates of mobilome evolution manifest as high between-clade heterogeneity at the scale of all ray-finned fishes. Clade-specific changes in the mobilome are readily apparent when considering a phylogenetic principal components analysis (PCA) of mobilome abundances (Fig. 5). For example, PC1 corresponds to sharp divergence between *Esox*, salmonids, and three bichirs, from acanthomorphs (Fig. 5 and supplementary figs. S4 to S6, Supplementary Material online). Some of this divergence is possibly explained by genome duplications specific to *Esox* and salmonids, suggesting future studies of the mobilome in these taxa to be of high research interest. Likewise, the hundreds of millions of years of independent evolution in bichir and holosteans lineages could also have eroded some signature of TE diversification. Our sampling includes all the extant lineages of holosteans and bichirs that comprise the deepest branches in their respective phylogenies, as such there are no known additional lineages that could be sampled to polarize ancestral state reconstructions of the mobilome at deeper divergences. Regardless, the alignment of taxa with PC1 values coincides with the prevalence of high percentage of DNA transposons that comprise the TEs within fish genomes that influence genome size variation among teleosts such as zebrafish (supplementary fig. S3, Supplementary Material online), medaka, stickleback, and *Tetraodon* (Jiang et al. 2016). It is certainly not possible to discount a possible role for life-history shifts shaping the mobilome among closely related species or that differences between class 1 and class II replications serve as an opportunity for substantial expansion of the mobilome.

## Conclusion

We find no evidence for a discernible temporal signature of TE diversification coincident with the TGD, supporting the hypothesis that this genome duplication event did not exert a long-lasting influence on TE diversification (Chalopin and Volff 2017). Our results suggest that the ray-finned fish mobilome appears to maintain a consistent profile, with substantial similarity between the mobilomes of teleosts and nonteleost ray-finned fishes. However, mobilome content between taxa varied substantially, from around 55% in Zebrafish to about 6% in pufferfish genomes. These shifts cannot be entirely explained by differences in genome size as genome compaction in smooth pufferfish like *T. rubripes* and *Tetraodon nigroviridis* is not associated with an overall reduction of retrotransposon diversity (Volff et al. 2003). Similarly, Medaka and Zebrafish have been found to have very similar L1 retrotransposon diversity, despite a large disparity in genome size (Kordis et al. 2006). Instead, these results strongly imply that the history of the teleost mobilome is one of lineage-specific evolution. In this way, the diversification of TEs may mirror the general dynamics of gene-birth found in multigene families in which there is consistent turnover of paralogs through time (Pasquier et al. 2016). If true, then the impact of ploidy events may be short-lived at best, and shifts in TEs may be more aligned with the concepts of hopeful monsters over deeper evolutionary scales.

Our analyses support the observation that early diverging aquatic vertebrates (e.g. coelacanth, ray-finned fishes, cartilaginous fish, and lamprey) contain a significantly broader spectrum of mobilome diversity in the genome relative to birds or mammals, ranging from 22 to 27 TE superfamilies. Among these, certain autonomous elements like ERVs, LINE1 retrotransposons, TcMariner, or hAT DNA transposons, as well as nonautonomous ones like V-SINE (Piskurek and Jackson 2011), are widely distributed across all the vertebrates. This suggests their likely presence in ancestral jawed vertebrate genomes. In contrast, we see a lower distribution of endoretroviruses (ERVs) in actinopterygians than mammals and birds, with the amount ranging from 0.033% in Fugu to 0.76% in zebrafish (Carducci et al. 2020). Our findings of clade-specific heterogeneity within ray-finned fishes, suggests that future studies between more closely related clades to be particularly fruitful as genomic resources become available. In particular, such studies could reveal superfamilies that have been lost or are on the path to extinction in specific lineages, similar to studies revealing such findings for L2 and Helitrons in birds (Chalopin et al. 2015) and gypsy retrotransposons in birds and mammals (Volff et al. 2003). It is clear that we are only beginning to unmask the complexity of transposon dynamics and decipher the intricate processes that contribute to TE diversification over deep evolutionary timescales.

## Methods and Materials

### Sample Acquisition and Sequencing

All research involving live animals was performed in accordance with relevant institutional and national guidelines and regulations, and was approved by the North Carolina State University Institutional Animal Care and Use Committee (IACUC). We acquired an individual *P. bichir* specimen (Yoder lab ID 0051) through the pet trade that was anesthetized using MS-222 for the extraction of blood (10 ml). The fish was then euthanized for dissection of tissue samples and the voucher specimen was deposited in the North Carolina Museum of Natural Sciences Ichthyology Collection (NCSM 111902). Blood was shipped to Dovetail Genomics, LCC (Scotts Valley, CA) for genomic DNA extraction, library preparation, sequencing, and assembly. Samples were extracted by Dovetail staff using Qiagen and Cell Culture Midi Kit (Qiagen, Gmbh), yielding DNA with an average fragment length of 95 kbp that was used in the construction of HiC sequencing libraries.

### Chicago Library Preparation and Sequencing

A Chicago library was prepared using the methods as described in Putnam et al. (2016). About 500 ng of High Molecular Weight genomic DNA, with mean fragment length of 95 kbp. Chromatin was reconstituted in vitro by incorporating the DNA with purified histones and chromatin assembly factors and then fixed by formaldehyde. *Dpn*II was used to digest the chromatin, followed by filling in the 5′ overhangs with biotinylated nucleotides, and then ligating the free blunt ends. After the ligations step, DNA was purified from protein by reversing the crosslinks. Biotinylated free ends were removed from the purified DNA by treating it. The DNA was sheared to ∼350 bp fragment size and Illumina compatible adapters along with NERNext Ultra enzymes were used to generate sequencing libraries. Streptavidin beads were used ahead of PCR enrichment of each library to isolate the biotin-containing fragments. The libraries were sequenced on an Illumina HiSeqX providing 30.58x physical coverage of the genome (1 to 100 kbp).

### Dovetail Hi-C Library Preparation and Sequencing

The preparation of a dovetail Hi-C library was executed as described (Lieberman-Aiden et al. 2009). For each library, the chromatin was fixed in place in the nucleus by crosslinking with formaldehyde, and the extracted fixed chromatin was then digested with the restriction enzyme *Dpn*II that produces 5′ overhangs. These 5′ overhangs were filled in with biotinylated nucleotides, and then the resulting blunt ends were ligated. Crosslinks were then reversed to obtain the purified DNA, and which was treated to remove excess biotin. A Hi-C library was then created by shearing the DNA to ∼350 bp mean fragment size. Sequencing libraries were generated using NEBNext Ultra enzymes and Illumina compatible adapters. Streptavidin beads were used prior to PCR enrichment of each library to isolate the biotin containing fragments. Libraries were sequenced using an Illumina HiSeqX which yielded 191 million paired end reads (2 × 150 bp) and provided 8,427.66 x physical coverage of the genome (10 to 10,000 kbp).

### Scaffolding the Assembly With Hi-Rise

Sequencing reads were assembled with Hi-Rise, a software pipeline designed to scaffold genome assemblies using proximity ligation data (Putnam et al. 2016). It uses the de novo assembly, shotgun reads, Chicago library reads and Dovetail HiC library reads as input and conducts an iterative analysis. The shotgun and Chicago library sequences are first aligned to the draft input assembly using a modified SNAP read mapper (http://snap.cs.berkeley.edu), with modifications such as masking out the base pairs that followed a restriction enzyme junction. Hi-Rise then modeled the separations of Chicago read pairs mapped within draft scaffolds, using a likelihood model for genomic distance between read pairs. The likelihood model produced by HiRise was used to identify and break putative misjoins and also score and make prospective joins. Dovetail HiC library sequences were analyzed using the same methods after aligning and scaffolding the Chicago data. Once all the sequences were aligned and scaffolded, shotgun sequences were used to close the gaps between contigs.

### Contamination Removal and Species Verification

Dovetail staff have noted that when pooled with other samples on Illumina sequencing platforms, 10X Chromium Genome solution libraries are susceptible to a small degree of index hopping that can result in minor incorrect assignment of sequenced reads during demultiplexing. This low level of index misassignment, typically results in sequence contaminants impacting, but limited to, small scaffolds (typically <10 kb) in the final assembly. To mitigate this, dovetail staff leveraged any uncharacteristic number of reads per barcode associated with impacted scaffolds to identify and reliably isolate them from the final assembly. This was accomplished by aligning the 10X reads to the supernova assembly, recording the barcode count for the aligned reads, and recording the number of reads that aligned to each scaffold and were tagged with the same barcode. The median number of reads per barcode for each scaffold were then calculated and scaffolds with a distinct anomalously low ratio were removed.

Currently, *P. bichir* is described as a single species with the subspecies *Polypterus bichir bichir* and *Polypterus bichir lapradei* no longer considered valid (Moritz and Britz 2019). This sinking of subspecies is a consequence of the high degree of morphological similarity (Britz and David Johnson 2003; Britz and Johnson 2010). However, molecular investigations have suggested the possibility that *P. bichir bichir* and *P. bichir lapradei* may be genetically distinct (Suzuki et al. 2010) and these have been treated as independent lineages (Near et al. 2014). As no genetic species delimitation analyses have been conducted between these putative genetic lineages, we extracted the mitochondrial barcode COI from our assembly and used a BLAST search against polypterid sequences on NCBI to verify identification that included barcodes from *P. bichir bichir* and *P. bichir lapradei*. This ensured that we accounted for possible future taxonomic revisions while remaining consistent with current taxonomy.

### RNA Sequencing and Assembly

RNA was extracted (Qiagen RNeasy kit) from the spleen, kidney, gill, heart, eyes, and intestine of the same individual *P. bichir* as the genome sequence. Quantity and integrity of the isolated RNA were assessed using a NanoDrop 1000 (Thermo Fisher) and Agilent Bioanalyzer, respectively. The process of mRNA enrichment was done using oligo(dT) beads, and rRNA was removed using a Ribo-Zero kit (Epicentre, Madison, WI). Each RNA extraction was equalized for a final concentration of 180 ng/µL. Library preparation and sequencing was performed by Novogen Corporation (Sacramento, CA). Next-gen sequencing (2 × 150 bp paired end reads) was performed on a NovaSeq 6000 instrument (Illumina). Adapter sequences and poor quality reads were filtered with Trimmomatic v34 (Bolger et al. 2014). The transcriptome was de novo assembled with Trinity v2.11.0 (Grabherr et al. 2011) followed by BUSCO analysis to assess completeness of the transcriptomes (Manni et al. 2021). Raw reads and computationally assembled transcriptome sequences were deposited onto NCBI under the accession numbers SRR19537224–SRR19537230 and GKOV01000000, respectively.

### Gene Ontology Assessment

We conducted a series of analyses for gene ontology assignment. First, the RNA-seq data assembled using Trinity was translated with Transdecoder, enabling the identification of potential coding regions in the transcript sequences. The longest open reading frames (ORFs) were extracted and subjected to BLASTx and BLASTp analyses against the Uniprot database (November 2021 release), yielding the top target sequences for each transcript. Subsequently, Hmmscan v.3.3.2 was employed to search for protein sequences in the Pfam-A database (November 2021 release). Signalp v.5.0b and TMHMM v.2.0c were used to detect signal peptides and transmembrane proteins, respectively. Trinotate v.3.2.2, in combination with the Trinity assembled transcriptome and the longest ORFs from Transdecoder, generated a gene transmap. This process facilitated the annotation of transcripts using Trinotate, followed by further analysis to obtain the Gene Ontology (GO) annotations. For visualization of the GO terms, the “enrichplot’ and “ggupset’ packages in R were employed. These steps collectively provided a comprehensive understanding of the functional attributes of the transcriptome data, to understand the underlying biological processes and pathways associated with the studied organisms (supplementary figs. S7 to S27, Supplementary Material online).

### Annotation and Genome Quality Assessments

The *P. bichir* genome annotation was done using the commonly used BRAKER2 (Brůna et al. 2021). To accomplish this, we first used RepeatModeler (Version 5.8.8) (Flynn et al. 2020) to model the repeats in the genome sequence. We then used RepeatMasker (Smit et al. 2005) to mask the repeats found with RepeatModeler and remove them from the genome. The masked genome and the transcriptome aligned using HISAT2 (version 2.1.0) (Kim et al. 2019) were then used to annotate the genome using the Genemark-ET option in BRAKER. The genemark.gtf file was subsequently used by Augustus (Gabriel et al. 2021) to model the proteins.

The completeness of the protein sequences was assessed using BUSCO v. (5.5.0) (Simão et al. 2015; Seppey et al. 2019) with both the Actinopterygii (actinopterygii_odb10, busco.ezlab.org) and vertebrate (vertebrata_odb10, busco.ezlab.org) databases. As polypterids are several hundred million years divergent from all other actinopterygians (Dornburg et al. 2014; Near et al. 2014), the use of the second vertebrate-wide database allowed us to verify similar levels of assembly completeness and mitigate against a potentially teleost-biased ray-finned fish database that may not capture loci in a deeply divergent taxon. We additionally conducted an analysis of synteny between our assembly of the *P. bichir* genome and the previously sequenced *P. senegalus* and *E. calabaricus* genomes using D-Genies (Cabanettes and Klopp 2018). To estimate the relative ages of TEs within the genome, we generated a Kimura distance plot using Repeatmasker with the -a parameter to get the alignment file (.align). This was analyzed using “calcDivergenceFromAlign.pl’ to get the divergence summary divsum file (.divsum), which was further analyzed using “createRepeatLandscape.pl’ to create the comprehensive repeat landscape.

### Comparative Analyses of the Actinopterygian Mobilome

To assemble a dataset of TE content across major ray-finned fish lineages, we integrated the results of the repeat analysis of the *P. bichir* genome with other publicly available analyses of TEs in ray-finned fishes. For nonteleosts, this captured TE content from two additional polypteriform genomes for *E. calabaricus* (Helfenrath et al. 2021) and *P. senegalus* (Fujito and Nonaka 2012), *A. ruthenus* (Du et al. 2020) as a representative of Chondrostei, as well as *Lepisosteus oculatus* (Braasch et al. 2016), *L. osseus* (Mallik et al. 2023), and *Amia calva* (Thompson et al. 2021) as representative holosteans. These data were integrated with data from 98 teleost genomes previously analyzed (Reinar et al. 2023), that capture the majority of major teleost lineages including Elopomorpha, Osteoglossomorpha, Otocephala, and a large number of acanthomorph and nonacanthomorph euteleosts. This study used the same RepeatModeller-based approach for TE identification as our methods above, making it appropriate to combine with our dataset. We generated a consensus sequence file using RepeatMasker to obtain detailed information TEs that were extracted from the resulting .out and .tbl files. This yielded a dataset of TE content for 105 ray-finned fish genomes. To place this data into a comparative phylogenetic framework, we first obtained a time calibrated phylogeny of all taxa from TimeTree v5 (Kumar et al. 2022). As this tree lacked resolution for acanthomorph lineages, we modified branch lengths to reflect the age estimates from a recent analysis of acanthomorph divergence times based on ultraconserved elements (Ghezelayagh et al. 2022) that is consistent with other published divergence time estimates of this superradiation (Near et al. 2013; Hughes et al. 2018). We additionally modified the topology to reflect the proposed sister relationship between Osteoglossomorpha and Elopomorpha based on genomic and transcriptomic sequence analyses (Hughes et al. 2018; Vialle et al. 2018; Hao et al. 2020; Wcisel et al. 2020).

We used the ggtree and ggtreeExtra packages (Yu 2022) in R 4.3.1 to visualize the distribution of TE abundances (LTR, LINE, SINE, DNA) across the evolutionary history of actinopterygians. We additionally used ggtree in conjunction with phytools (Revell 2012) to visualize genome size and TE content variation between species alongside a likelihood based ancestral state estimation of changes in genome size across the phylogeny conducted in phytools. To assess if changes in genome size were correlated with the changes in the overall abundance of TEs, or changes in the abundances of LTRs, LINEs, SINEs, or DNA elements, we conducted a series of phylogenetic linear regressions using the phylolm package in R. Model fits were assessed by quantification of Akaike Information Criterion (AIC) scores (supplementary table S4, Supplementary Material online) Regressions were conducted under a Brownian model of trait evolution (supplementary table S6, Supplementary Material online). To assess whether results were robust to the underlying model of character evolution, analyses were repeated using an Ornstein–Uhlenbeck (OU) model of character evolution (supplementary table S7, Supplementary Material online). A similar set of phylogenetic regressions was next conducted to assess if increases in the abundance of elements (e.g. LINEs, SINEs, etc.) were positively correlated with each other, or if negative correlations exist, allowing us to assess whether elements have antagonistic evolutionary dynamics (supplementary table S8, Supplementary Material online). Next, we performed a set of regressions assessing whether changes in the abundances of elements were correlated with changes in maximum body size, latitude, or maximum depth of occurrence. Body size and depth data for each species were taken from fishbase using the rfishbase package in R (Boettiger et al. 2012). Latitudinal data were calculated using occurrence data from the Global Biodiversity Information Facility (gbif) using rgbif v3.7.8. Although imperfect, both BM and OU models are commonly used models for comparative analyses of the above traits (Iglesias et al. 2015; Medeiros et al. 2022; Parker et al. 2022), with OU models often providing a better fit for depth data depending on the temporal scale. This fit may be a consequence of clade specific expansions into different depth zones occurring over deep time scales. We further tested for differences in TE content between teleosts and nonteleosts using a phylogenetic ANOVA in phytools phylANOVA with multiple comparison correction using the Benjamini–Hochberg procedure (Benjamini and Hochberg 1995).

To reconstruct the ancestral mobilome of early ray-finned fishes, we focused on the 25 elements most commonly studied in analyses of ray-finned fish TEs. The presence/absence data were scored for each species and each element and used as input data for a model averaged stochastic character mapping approach (Bollback 2006) implemented in phytools. This approach expands the standard phytools implementation to ancestral state estimation by allowing possible models of character change (e.g. “Equal Rates”, “All Rates Different”) in phytools to contribute to the reconstruction in relation to their Akaike weight. We assessed the degree to which variation in TE content could be explained by evolutionary history through quantification of the phylogenetic signal of overall TE abundance as well as the abundances of each TE type. This was accomplished using the phylosig function in phytools to calculate both Pagel's λ (Pagel 1999) and Blomberg et al.'s *K* (Blomberg et al. 2003) Values of λ are distributed between zero and 1, with a zero value representing the absence of phylogenetic signal and a value of 1 corresponding to the expectations of Brownian motion on a phylogeny. To assess statistical significance, we compared our empirical *λ* values to the null expectation that *λ* = 0 for each trait via a likelihood ratio test. Blomberg's *K* compliments estimates of *λ*, with values <1 indicating less phylogenetic signal than would be expected given a model of Brownian motion, and value >1 indicating a higher than expected coupling between the distribution of traits and the underlying phylogeny. To assess statistical significance of *K*, we compared our empirical *K* values to null distributions of expected *K* values based on 10,000 permutations of each trait on the phylogeny.

To visualize the major axes of variation of the ray-finned fish mobilome, we conducted a phylogenetic principal component analysis (pPCA) using the phyl.pca function in phytools. The resulting PC axes were then used with the phylomorphospace function in phytools to project the phylogeny into the resulting PCA space using ggplot2. Additionally, we conducted a pPCA on the abundances of the subtypes of all TEs based on the amounts derived from the .out files. As preliminary analyses indicated a correlation between elements and overall genome size, pPCAs were repeated on the residuals resulting from a regression of genome size versus TE content, mirroring similar approaches to accounting for traits that covary with another trait (e.g. limb proportions and body size, etc.).

## Supplementary Material

evae272_Supplementary_Data

## Data Availability

The *P. bichir* genome sequence is available through NCBI Bioproject PRJNA811142. Raw transcriptome reads are available through NCBI under the accession number SRR19537224, SRR19537225, SRR19537226, SRR19537227, SRR19537228, SRR19537229, SRR19537230. Computationally assembled transcriptome sequences are available on NCBI under the accession number GKOV000000000. All the files and code used for TE analysis and visualization are available on Zenodo (DOI: 10.5281/zenodo.10398557).
