# Supplementary material for: Investigating the Impact of Whole-Genome Duplication on Transposable Element Evolution in Teleost Fishes
Source: Genome Biol Evol. 2024 Dec 23;17(1):evae272. doi: 10.1093/gbe/evae272 (PMC11785729; doi:10.1093/gbe/evae272)
Supplement: evae272_Supplementary_Data [file evae272_supplementary_data.zip › Table S7.pdf]

Supplemental Table S7. Results of phylogenetic linear models evaluating the correlation between TE abundance and genome size, latitude, body size, or depth under an OU model.

Trait	TE	Intercept (a)	Coefficient (b)	SE (b)	T Value	P Value
Genome size	All	20.22	1.15e-08	1.79e-09	6.42	**4.55e-09**
	DNA	4.76	4.15e-09	7.44e-10	5.58	**2.05e-07**
	LINE	1.45	3.21e-09	4.24e-10	7.58	**1.75e-11**
	LTR	0.09	1.01e-09	2.23e-10	4.55	**1.52e-05**
	SINE	0.04	2.51e-10	1.14e-10	2.21	**2.95e-02**
Latitude	All	1.52e-05	-5.62e-03	0.06	-0.09	0.93
	DNA	7.89	1.24e-03	0.02	0.05	0.96
	LINE	4.66	-2.73e-02	0.01	-1.79	0.07
	LTR	1.67	1.60e-04	0.01	0.02	0.98
	SINE	0.77	-6.45e-03	0.01	-1.93	0.06
Body Size	All	29.69	-0.19	1.03	-0.19	0.85
	DNA	11.18	-0.89	0.40	-2.20	**0.03**
	LINE	2.04	0.51	0.25	2.01	**0.05**
	LTR	0.59	0.29	0.11	2.56	**0.01**
	SINE	0.32	0.07	0.06	1.29	0.19
Depth	All	27.89	1.813e-03	1.13e-03	1.61	0.11
	DNA	7.24	1.50e-04	3.72e-04	0.40	0.69
	LINE	3.45	2.26e-04	3.14e-04	0.72	0.47
	LTR	1.58	4.3e-05	1.52e-04	0.28	0.78
	SINE	0.57	2.38e-05	8.11e-05	0.29	0.77

Bolded values indicate *P*-values below 0.05.
